# The impact of genomics on precision public health: beyond the pandemic

**DOI:** 10.1186/s13073-021-00886-y

**Published:** 2021-04-23

**Authors:** Muin J. Khoury, Kathryn E. Holt

**Affiliations:** 1 Office of Genomics and Precision Public Health, Centers for Disease Control and Prevention, Atlanta, GA USA; 2 Monash University, Melbourne, Australia; 3 London School of Hygiene and Tropical Medicine, London, UK

Precision public health has been defined in many ways [1]. It can be viewed as an emerging multidisciplinary field that uses genomics, big data, and machine learning/artificial intelligence to predict health risks and outcomes and to improve health at the population level. Just like precision medicine seeks to provide the right intervention to the right *patient* at the right time, the aim of precision public health is to provide the right intervention to the right *population* at the right time, with the goal of improving health for all.

Genomic technologies have been at the leading edge of applications in clinical medicine and have the potential to revolutionize public health. We are pleased to introduce this special issue of *Genome Medicine* on the impact of genomics on precision public health, which highlights the utility of genomic tools in public health research and practice in the fight against communicable and noncommunicable diseases. This is particularly timely, given the battle against the COVID-19 pandemic, which has necessitated the application of genomic approaches to track the origin, transmission and evolution of the SARS-CoV-2 virus globally, as well as to understand differential host susceptibility, response, severity, and outcomes. Beyond genomics, granular data from population surveillance approaches are being used to target public health interventions. In addition, big data, digital technologies, and mobile health applications have been instrumental in defining the natural history of COVID-19 and identifying prognostic factors through machine learning and artificial intelligence [2].

## Human genomics and public health

Genomics plays an emerging role in clinical and public health research. An increasing number of genomic tests are available in practice, such as tumor genome analysis for targeted treatment, non-invasive prenatal screening, and genomic tests for childhood and rare disorders. More generally, public health research is needed to assess how genomics fits in an overall ecological model of health that takes into account the combinations of genes and environmental, behavioral, and social determinants [3]. As many common chronic diseases have known environmental, social, and behavioral risk factors (e.g., smoking, physical activity, diet, racial, ethnic and economic factors, and access to health care), it is important to evaluate the benefits, harms and costs of the use of human genomic information in the prevention and control of these diseases. Applying genomic tools in practice will require a multidisciplinary research collaboration that includes epidemiologists, behavioral, social, and communication scientists, health services researchers, and many others [3].

A barrier to using human genomic information for improving population health is poor uptake of evidence-based interventions and the potential for widening existing health disparities. Fields such as implementation science will be needed to identify the most effective methods and strategies for integrating the use of genomic applications in population health. Partnerships between healthcare organizations and public health programs can help bridge the implementation gap and reduce health disparities.

To date, newborn screening for treatable inherited conditions represents the most successful human genomic application in public health, but population screening across the lifespan for other genetic conditions is increasingly possible. Abul-Husn et al. report in this issue that the addition of genomic conditions with high prevalence in non-European populations to a genomic screening program increases the number of non-European participants choosing to receive results [4]. In addition, Stranneheim et al. integrate whole genome sequencing into the Stockholm-area healthcare system to aid rare disease diagnosis, making important strides in clinical-academic partnerships [5].

Other recent studies further highlight the impact of public health programs in accelerating the identification of individuals with hereditary cancers in populations (e.g., Lynch syndrome, hereditary breast and ovarian cancer) [6]. In order to achieve this, public health activities must involve the identification of people at risk in health systems and through testing relatives of affected individuals, known as cascade screening. Public health programs can also help monitor implementation of genomic medicine, quantify health disparities in implementation, and develop approaches to address them.

Other emerging applications include genetic predisposition to adverse drug effects (pharmacogenomics), carrier testing of prospective parents, and use of polygenic risk scores (PRS) in disease detection and prevention. For example, Isgut et al. show that PRS improve cardiovascular risk stratification early in life when later-life risk factors are unknown. By middle age, when many risk factors are known, improvement attributed to PRS is marginal for the general population [7]. Even in the genomics age, a simple family health history assessment can enhance the delivery of precision medicine in health care and population settings [8]. Moreover, as millions of people have sought direct-to-consumer genetic tests, public health programs can help educate the general public about the promise and limitations of emerging tests in improving health, as well as to track the impact of genomic tests at the population level.

Of note, the special issue presents articles on the ethical implications of the use of genomics in public health in the COVID-19 era. Lewis and Green [9] discuss how most of the ethical, legal, and social issues (ELSI) that apply to single gene diseases—such as the relevance of results to family members, the approach to secondary and incidental findings, and the role of expert mediators—are also relevant in the applications of PRS in practice.

Geller et al. [10] review the ethical considerations and implications of ongoing genomic studies that assess host factors associated with variability in susceptibility, infectivity, and disease severity in patients with COVID-19, or those exposed to it. Juengst et al. [11] propose a new ethical framework to ensure that the benefit of precision public health interventions based on advances in genomics research is not outweighed by the risks they pose to individuals, families, and vulnerable segments of the population.

## Infectious disease genomics and public health

Genomics has a similarly important role to play in understanding and managing infectious disease at both the individual and population levels, as it can be used to gain precision with respect to both the pathogen and the infected host population.

On the pathogen side, whole genome sequencing (WGS) is fast becoming the standard assay for characterizing infectious diseases and is potentially an incredibly rich source of information to guide public health interventions [12]. For example, pathogen genomics is now widely used for the investigation of foodborne or hospital outbreaks, and it is increasingly incorporated into surveillance for clinically important features such as vaccine antigens (to inform vaccine composition) and antimicrobial resistance determinants (to inform treatment). Genomics is also increasingly applied for pathogen detection and diagnostics in the clinic. In this context, DNA sequencing can increase the precision of diagnosis beyond species to the identification of lineages or variants that may be associated with different degrees of risk and thus prompt different responses in terms of clinical management of the individual patient (e.g. antimicrobial treatment choice) or public health management (e.g. triggering infection control or contact tracing).

Infection is a two-way street, and functional genomic characterization of the host response can also have diagnostic and prognostic utility, providing insight into the nature and extent of immunological responses and helping to identify appropriate treatments for different patient groups. For example, Aschenbrenner et al. studied transcriptomes of COVID-19 patients [13], yielding insights into the natural history of disease and highlighting differences in the immune response of patients with severe vs mild disease. These specific response signatures were probed to identify potential drugs for therapeutic repurposing, targeted specifically to those with severe disease. Being able to more precisely define which host populations are most at risk of infection through genomic risk prediction is also an attractive proposition that could have significant impacts on population health by allowing preventative measures to be targeted to the most at-risk subgroups, although this comes with the same ethical concerns as the genomic prediction of non-communicable disease risk and in fact could intersect with it. In this special issue, Kachuri et al. [14] identified genetic variants associated with antibody responses to antigens for 16 different viruses, many of which were also associated with non-communicable diseases including cancer.

The COVID-19 pandemic has stimulated the refinement of methods for rapid high-throughput pathogen sequencing, such as the CoronaHIT method for SARS-CoV-2, published in this special issue [15], and illustrates some of the ways that real-time pathogen genome data can be harnessed to inform public health strategy in the context of an ongoing epidemic. Genomics is the primary method for monitoring variation in pathogen populations and identifying variants that may be of public health concern due to (i) increased transmissibility; (ii) increased severity; (iii) differences in sensitivity/specificity of current diagnostic tests; and (iv) escape from interventions, such as host immunity (vaccine-induced or natural) or sensitivity to drugs. Identification of such variants can increase precision of the public health response, by targeting scarce resources towards the most concerning variants, for example community-based testing in areas of England where importation of potential vaccine-escape variants of SARS-CoV-2 were detected, or updating diagnostics and vaccines to ensure coverage of the continually evolving pathogen population. In addition, phylogenomic analyses can be used to track transmission of the virus at different spatial scales, such as assessing the contribution of travel-associated strain introductions to establishment of the epidemic in a particular country [16] or investigating within-hospital transmission [17]. The pandemic has also highlighted challenges associated with generating and sharing sequence data in a timely, equitable, and ethical manner, as well as challenges around data visualization, naming of genetic variants, and communicating results both within the public health sector and with the wider public.

It has been suggested that the COVID-19 pandemic may be amplifying another major global health crisis, that of antimicrobial resistance [18]. Two articles in this special issue illustrate the important role of genomics in understanding and controlling the burden of antimicrobial resistant infections in hospitals. Berbel Caban et al [19] present a method to facilitate the detection of in-hospital spread of methicillin resistant *Staphylococcus aureus* (MRSA or “golden Staph”) by combining genomic and epidemiological data. The genetics of antimicrobial resistance can be incredibly complex, and for most pathogens, the overall burden of resistant infections is the combined result of the spread of resistance genes and plasmids between bacterial strains and the transmission of bacterial strains between patients. Arredondo-Alonso et al [20] use genomics to disentangle and quantify these contributing factors, which could help to focus infection control efforts in future.

## Looking ahead

The field of precision public health is still in its infancy and genomics will be a major contributor to its growth in the next decade. The COVID-19 pandemic provides both a challenge and an opportunity for further evolution of precision public health, as new tools and technologies are beginning to complement traditional public health approaches. In the next decade, additional developments in the field will require leadership and commitment to enhance data collection and coordination across geographic boundaries, harmonization and integration of different types of data beyond genomics, and robust assessment of ethical, legal, and social implications of new technologies for the benefits of humanity worldwide. Many of these issues are currently being tackled by the recently formed Public Health Alliance for Genomic Epidemiology [21], which aims to improve openness, interoperability, accessibility, and reproducibility in public health microbial informatics globally. As the COVID-19 pandemic clearly shows, we need the tools of genomics more than ever before in the fight against infectious diseases. Beyond fighting outbreaks, genomics will become an essential component of public health in the twenty-first century for communicable and noncommunicable diseases.
